# Cellular senescence-like features of lung fibroblasts derived from idiopathic pulmonary fibrosis patients

**DOI:** 10.18632/aging.100807

**Published:** 2015-09-22

**Authors:** Hagai Yanai, Albert Shteinberg, Ziv Porat, Arie Budovsky, Alex Braiman, Rolf Zeische, Vadim E. Fraifeld

**Affiliations:** ^1^ The Shraga Segal Department of Microbiology, Immunology and Genetics, Center for Multidisciplinary Research on Aging, Ben-Gurion University of the Negev, POB 653, Beer Sheva 84105, Israel; ^2^ Flow Cytometry Unit, Department of Biological services, Weizmann Institute of Science, Rehovot 76100, Israel; ^3^ Judea Regional Research & Development Center, Carmel 90404, Israel; ^4^ Division of Pulmonary Medicine, Department of Internal Medicine II, Medical University of Vienna, Waehringer Guertel 18-20, 1090 Vienna, Austria

**Keywords:** cellular senescence, idiopathic pulmonary fibrosis, fibroblasts, myofibroblasts, aging

## Abstract

Idiopathic pulmonary fibrosis (IPF) is an age-related fatal disease with unknown etiology and no effective treatment. In this study, we show that primary cultures of fibroblasts derived from lung biopsies of IPF patients exhibited (i) accelerated replicative cellular senescence (CS); (ii) high resistance to oxidative-stress-induced cytotoxicity or CS; (iii) a CS-like morphology (even at the proliferative phase); and (iv) rapid accumulation of senescent cells expressing the myofibroblast marker α-SMA. Our findings suggest that CS could serve as a bridge connecting lung aging and its quite frequent outcome -- pulmonary fibrosis, and be an important player in the disease progression. Consequently, targeting senescent cells offers the potential of being a promising therapeutic approach.

## INTRODUCTION

It was Élie Metchnikoff who first proposed that “aging is the replacement of noble elements by ignoble ones” [1]. More specifically, he suggested that aging results from a progressive replacement of specialized tissues/cells by connective tissue/fibroblasts. Nowadays, tissue fibrosis is considered a major cause of progressive organ failure in aging and to be involved in numerous chronic age-related pathologies [2]. The lungs are among the organs most susceptible to excessive fibroproliferative processes, with a strong predisposition to fibrosis with advancing age [2]. One of the most aggressive and enigmatic manifestations of dysbalanced fibroproliferative repair is a disease called Idiopathic Pulmonary Fibrosis (IPF) [3-5].

IPF is a progressive lung disease of indefinite etiology in which the pathological hallmark is the heterogeneous alveolar and peribronchial accumulation of scar tissue in the lungs of affected individuals, dominated by an exaggerated non-regenerative repair called “usual interstitial pneumonia” (UIP) [6-8]. The incidence and prevalence of IPF increase almost exponentially with each decade of life; two-thirds of IPF patients are older than 60 years at the time of presentation, with a mean age of 66 years at the time of diagnosis [9]. Apart from the epidemiological observations, there is also evidence for mechanistic links between IPF and aging, and IPF is likely to share common pathophysiologic mechanisms with normal lung aging [5, 8, and 10]. Thus, IPF can be defined as an age-related disease. Neither mechanisms of IPF, nor effective pharmacological therapies have been established to date [4, 11, and 12]. The only effective treatment currently available for progressive lung fibrosis is lung transplantation [2].

IPF is marked by an accumulation of fibroblasts and myofibroblasts along with excessive production of type I collagen rich matrix [13]. As such, several features of fibroblasts derived from IPF patients have thus far been described, including desensitization to type-I-collagen-matrix-induced cell death [14], an increased invasiveness [15], reduced FOXO3a function [13], deficient *in vitro* repair response [16], resistance to apoptosis [17], an altered growth rate, and expression of tissue inhibitor of metalloproteinases (TIMPs) [18].

Recently, the pathogenesis of IPF was linked to the phenomenon of cellular senescence (CS) [8] – an acknowledged hallmark of organismal aging [19]. An extensive study on IPF lungs pinpointed to the presence of fibroblasts expressing CS biomarkers in fibroblastic foci [8]. CS markers have also been found in the overlying alveolar epithelial cells of IPF lungs [8, 20].

Given the importance of fibroblasts in the pathogenesis of IPF, we further address the issue by characterizing fibroblasts derived from lung biopsies of IPF patients in terms of proliferation rate, morphology, and expression of α-SMA (a myofibroblast marker), with a special focus on the connection of these characteristics to replicative and stress-induced CS. Here, we show that IPF fibroblasts are predisposed to CS and acquire some CS-like features far before reaching cell growth-arrest.

## RESULTS

### Replicative cellular senescence

**Figure 1:**
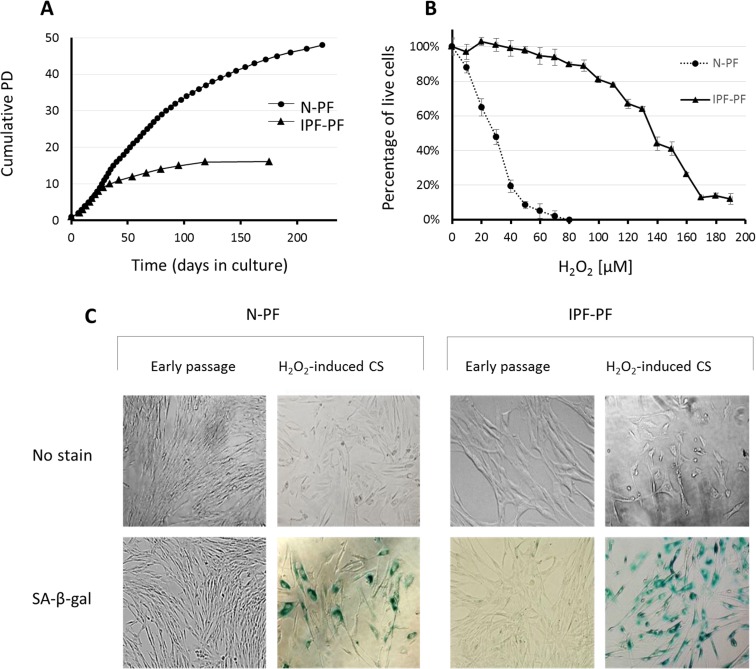
Growth curves and response to oxidative stress of IPF-derived and normal human pulmonary fibroblasts (**A**) Lung fibroblasts derived from IPF patients (IPF-PF) and healthy subjects (N-PF) during routine culture. PD stands for Population Doublings. Note that IPF-derived fibroblasts cease to proliferate after passage 16, whereas the N-PF ones are still in the logarithmic phase of cell growth. The difference between N-PF and IPF-PF growth curves is highly significant (Mann–Whitney *U*-test; p < 0.001). (**B**) Lung fibroblasts derived from IPF patients (IPF-PF) and healthy (N-PF) were treated with indicated doses of H_2_O_2_ for two hours, and tested for viability by Neutral Red assay (LD_50_ was 28.7 μM and 136 μM for N-PF and IPF-PF, respectively; p < E-06). Results represent 3 independent experiments. (**C**) SA-β-gal staining of normal (left panel) and IPF-derived fibroblasts (right panel) treated with H_2_O_2_ (the doses that caused 20% cytotoxicity were used).

While initially replicating in a rate similar to normal human pulmonary fibroblasts (N-PF), regularly passaged IPF-derived pulmonary fibroblast (IPF-PF) cultures reached CS much faster than N-PF (Fig. 1A). A clear slowing-down of the cell growth rate in the IPF-PF cultures was observed from passage 9-10, without any noticeable proliferation after passage 16. In contrast, N-PF continued to proliferate, displaying only a slight decrease in the cell growth rate at the time when the IPF-derived fibroblasts fully ceased to proliferate. Thus, the IPF-derived fibroblasts exhibit accelerated replicative CS.

### Oxidative stress-induced cellular senescence

The IPF-PF cells were found to be much more resistant to oxidative stress caused by H_2_O_2_ than N-PF (Fig. 1B). LD_50_ calculated from the dose-response curves was 28.7 μM and 136 μM for N-PF and IPF-PF, respectively, indicating almost a 5-fold increase in the resistance of IPF-PF vs. N-PF to H_2_O_2_. The doses of H_2_O_2_ that caused cytotoxicity of approximately 20% induced premature CS in both cell types, as evidenced by cessation of cell growth, morphological changes, and positive staining with SA-β-gal (Fig. 1C). This dose was over 6 times higher for IPF-PF (100 μM) compared to N-PF (15 μM). Thus, lung fibroblasts derived from IPF patients are much more resistant to oxidative-stress-induced cytotoxicity or CS than normal lung fibroblasts.

### Cell morphology

**Figure 2:**
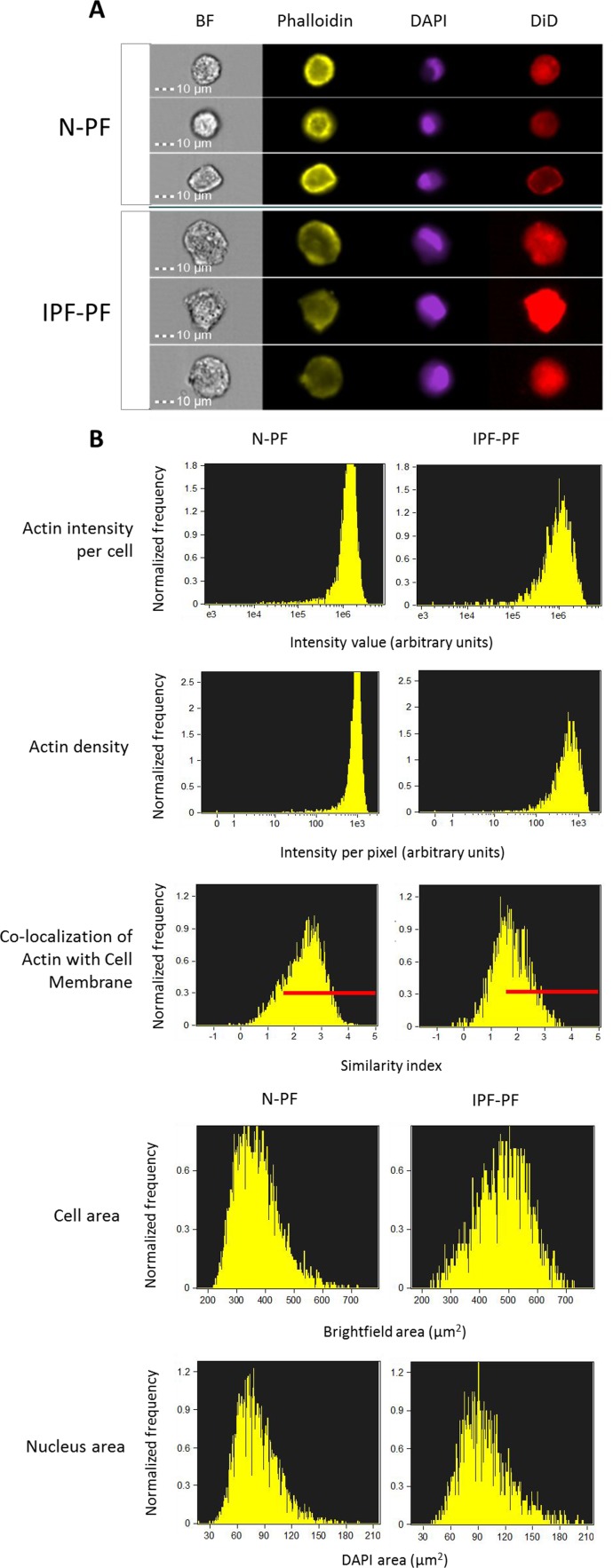
Morphology analysis of lung fibroblasts by ImageStreamX BF – bright field. Phalloidin, DAPI and DiD refer to staining of actin, nucleus and cell membrane, respectively. (**A**) Selected representative images. (**B**) Distribution of cell populations according to indicated criterion. The meaning of each variable is explained next to the images. Red lines indicate the cells in which actin is co-localized with cell membrane (score > 1.5). Note: IPF-PF vs. N-PF represent a more heterogeneous (more variable) cell population as evident by CV values (Table 1).

As seen in Fig. 1C, the morphology of IPF-PF cultures of early passages differs from that of N-PF, although at this stage they display the same growth rate. With this in mind, we further compared the morphology of N-PF and IPF-PF using ImageStreamX analysis, a highly efficient FACS-based single-cell technology [21]. The analysis revealed that IPF-derived fibroblasts display a distinct morphology from N-PF cells (Fig. 2, Table 1): (i) they were larger and had a more irregular shape; (ii) actin density was much lower and there were more cells with a weaker co-localization of actin with the cell membrane; (iii) the vast majority of morphological characteristics of IPF-PF were of markedly higher variability.

**Table 1:** ImageStreamX quantitative analysis of IPF-derived (IPF-PF) and normal human pulmonary fibroblasts (N-PF). All differences between N-PF and IPF-PF are highly significant (p < 0.001)

Component	Measurement	N-PF				IPF			
		Median	Min	Max	CV (%)	Median	Min	Max	CV (%)
Cell	Cell Area (μm^2^)	360	219	722	19.6	487	232	727	17.6
	Circularity index	15.4	3.8	45.8	37.3	13.4	3.6	46.5	40.9
	Width/length ratio	0.89	0.64	0.99	7.24	0.87	0.63	0.99	8.91
Nucleus (DAPI staining)	Nucleus Area (μm^2^)	77.8	17.8	194	27.0	93.5	37.8	206	27.6
	Nucleus Area/Cell Area	0.22	0.05	0.42	21.0	0.20	0.09	9.39	24.4
	Nucleus Polarity index	0.10	0.002	0.37	46.6	0.09	0.001	0.30	50.0
Actin (Phalloidin staining)	Total intensity index	1.3×10^6^	1,471	3.4×10^6^	36.6	0.98	1,797	3.7×10^6^	59.6×10^6^
	Intensity index (per pixel)	924	0	1,800	31.0	568	0	1,746	54.8
	Max Contour Position index	0.50	0	0.75	28.1	0.50	0	0.73	28.6
	Area of top 50% intensity index	114	0	364	46.4	152	0	412	50.8
	Co-localization index with membrane (DiD staining)	2.43	–0.41	4.32	29.5	1.70	–0.42	3.69	36.1

**Figure 3:**
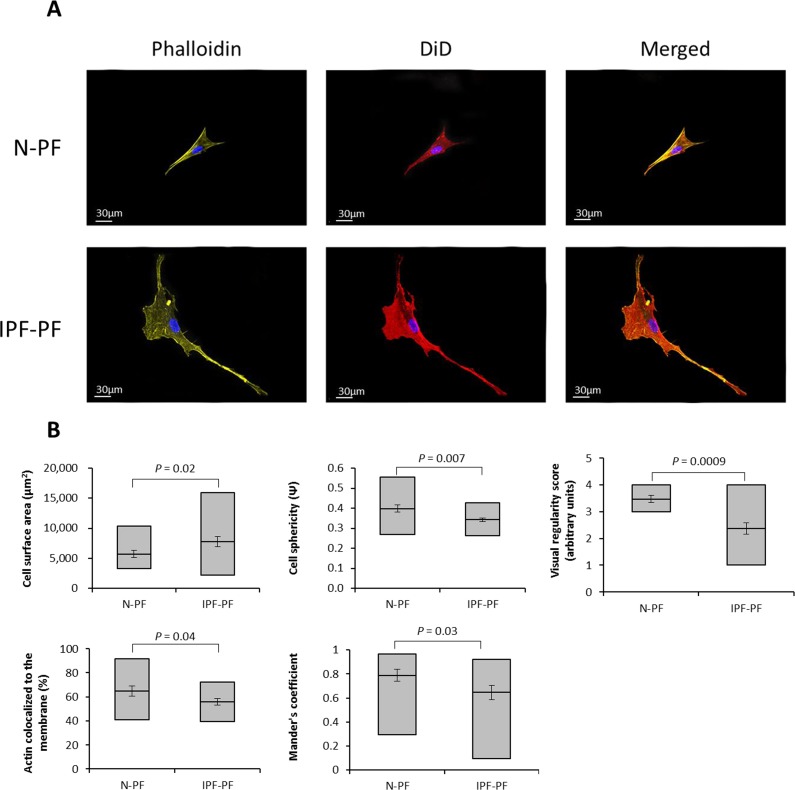
Morphology analysis of lung fibroblasts by Z-stack confocal microscopy (**A**) Representative image of lung fibroblasts derived from IPF patients (IPF-PF) and healthy (N-PF). Background subtracted. Phalloidin stains for actin; DiD for membrane and all pictures show DAPI staining of the nucleus (**B**) Quantification of cell morphology using the Imaris software (see methods) of confocal z-stack images taken for fibroblasts of early passage (passage 7). Whiskers indicate standard error [; bars indicate range (min/max); middle line indicates mean.

It should be noted that ImageStreamX measures cells in suspension. Considering the impact of cell attachment to its morphology, we further verified our quantifications by confocal Z-stack microscopy. As seen in Fig. 3, the N-PF cells of an early passage are relatively small and have a spindle-like shape, with actin strongly localized with the cell membrane (Fig. 3A, upper panel). In contrast, the IPF-PF cells of the same passage are much larger (median cell area of 2574 μm^2^ vs. 1893 μm^2^ for IPF-PF and N-PF, respectively), with irregular shape and a diffused distribution of actin within the cell (Fig. 3A, lower panel). Quantitative evaluation of the above descriptors is shown in Fig. 3B. Overall, these results correspond well with those obtained by the ImageSreamX analysis indicating that even actively proliferating IPF-PF cells display CS-like morphological features.

### Expression of α-SMA in senescent lung fibroblasts

**Figure 4:**
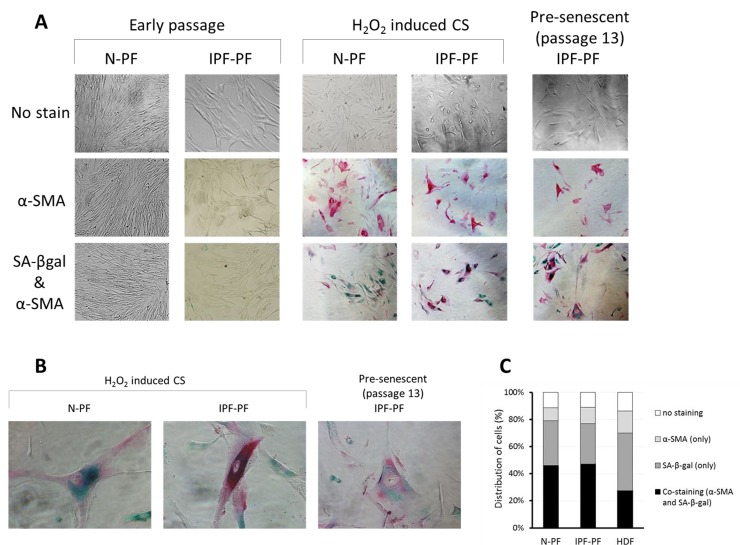
Immunostaining for SA-β-gal and α-SMA in primary cultures of pulmonary fibroblasts (**A**) early passages (upper left panels) and at cellular senescence (upper right panels). SA-β-gal – green staining; α-SMA – red staining. (**B)** Representative co-stained cells. (**C**) Distribution of cells expressing α-SMA, SA-β-gal, or both in senescent primary cultures of normal pulmonary (N-PF), IPF-derived fibroblasts (IPF-PF) and normal dermal Fibroblasts (HDF). The difference between the two pulmonary cell types was insignificant (p > 0.05).

Due to the pivotal role of myofibroblasts in IPF, we examined if they are presented in the primary cultures of IPF-PF, by detecting the expression of α smooth muscle actin (α-SMA), a known marker of myofibroblasts. Lung fibroblasts of early passages derived from both healthy donors and IPF patients showed no immunostaining for α-SMA (Fig. 4A). However, α-SMA-positive cells accumulated in CS cultures (Fig. 4A). Remarkably, co-staining with SA-β-gal clearly showed a high expression of α-SMA in senescent fibroblasts, both for IPF-PF and N-PF (Fig. 4), and this co-expression was higher for pulmonary fibroblasts than for dermal ones (Fig. 4B).

## DISCUSSION

Aging has a significant impact on wound healing [22] and could be a predisposing factor for the development of tissue fibrosis, especially in the lungs [2, 7, 8, 20, and 23]. In particular, IPF is clearly associated with advanced age and is therefore mostly observed in the elderly [2, 7, and 8]. In adult mammals, including humans, fibroblasts and myofibroblasts in particular, are the central cellular players in tissue remodeling after injury [24] and, as such, are a key driving force behind progressive fibrosis [8, 23, and 25].

The phenomenon of cellular senescence (CS) has recently been implicated in the pathogenesis of age-related diseases [26], IPF included [8]. CS is a state of irreversible growth arrest which is commonly accompanied by cell hypertrophy, an increased metabolic activity, high heterogeneity of cell morphotypes, increased resistance to apoptosis and secretion of pro-inflammatory substances [26-29]. Initially discovered in primary cultures of human fibroblasts, CS has also been observed *in vivo*, both for fibroblasts and other cell types, and the amount of senescent cells was found to increase with age [reviewed in 26, 30]. Further strengthening the mechanistic links between CS and IPF are observations on defects in telomere maintenance with subsequent telomere shortening (a well-known factor of CS induction) in IPF [31-37, and references therein].

In order to receive further insight into the links between CS and IPF, we investigated pulmonary fibroblasts derived from IPF patients, in light of well-established features of senescent cells. We found that in comparison to normal human pulmonary fibroblasts, the IPF fibroblast cultures displayed (i) some CS-like morphological features (e.g. enlarged cell and nucleus size, irregular cell form, heterogeneity of cell morphotypes) even in the proliferating stage, and also (ii) a high resistance of proliferating cells to oxidative stress-induced CS and cytotoxicity; (iii) despite this, the IPF fibroblasts showed an accelerated entry to replicative CS accompanied by an accumulation of senescent cells with features of myofibroblasts (high expression of α-SMA).

Provided that cells of patients retain, at least in part, their features also in *in vitro* conditions, our findings together with recent works in the field, could shed light on the progressive course of IPF and why aging is a predisposing factor for pulmonary fibrosis. First, although the IPF cells of early passages proliferated at the same rate as normal cells and did not stain for the CS marker SA-β-gal (or the myofibroblast marker α-SMA), they exhibited some morphological features similar to senescent cells such as cell hypertrophy and irregular morphology, as well as resistance to death stimuli. In other words, this could indicate that IPF fibroblasts are predisposed to CS, *long before* they differentiate into myofibroblasts. The observed changes in cell size and shape could in part be attributed to a low density of actin and its weak co-localization with the cell membrane (Fig. 2,3 and Table 1), a feature which may also be considered characteristic of senescent cells (data not shown). Since the lungs experience permanent mechanical insults [38], the proper organization of actin cytoskeleton in pulmonary cells is of utmost importance for their integrity and proper functionality. In view of this notion, the decreased levels of actin and its redistribution within the IPF fibroblasts may be an important factor in the course of the disease.

Second, as IPF is a disease of the elderly, the origin of IPF fibroblasts was obviously patients at an advanced age. These cells were more resistant to stress (H_2_O_2_)-induced cell death (see Fig. 1) than normal fibroblasts derived from the young. Similarly, Huang et al. [17], utilizing a mouse model of bleomycin-induced pulmonary fibrosis, observed that fibroblasts from the lungs of old mice, displayed a stronger fibrotic response and were more resistant to H_2_O_2_-induced apoptosis than those from young mice [17]. The resistance to stress-induced cell death of IPF fibroblasts may result in accumulation of damaged cells that otherwise would be eliminated. This, in combination with the observation that IPF fibroblasts were prone to enter replicative CS (see Fig. 1), may lead in the long run to an accumulation of senescent cells in areas of increased proliferation such as the wound repair site or the fibroblast foci in IPF. This accumulation was, in fact, observed *in vivo* by Hecker et al. [8].

Third, we found that α-SMA-positive cells accumulate in senescent fibroblast cultures, in line with the observation by Hecker et al. (2014) [8], and this accumulation was more evident in pulmonary fibroblasts than in dermal fibroblasts (Fig. 3). Of note, both senescent cells and myofibroblasts promote a pro-inflammatory microenvironment by secreting an increased amount of cytokines [for review see 28]. Yet, senescent cells and myofibroblasts have supposedly opposite effects on the extracellular matrix (ECM). While senescent cells are considered to downregulate the production of ECM proteins and upregulate ECM-degrading enzymes [39], myofibroblasts produce high level of collagen and promote extracellular matrix deposition [40]. Further complicating this issue is that senescent myofibroblasts may play a different role in acute and chronic fibrosis. For example, the induction of CS restricts the acute fibroproliferative response to injury in experimental hepatic fibrosis [32] and cutaneous wound healing [41]. However, while the induction of CS could be beneficial in the short run, a failure to eliminate CS cells and senescent myofibroblasts in particular appears to counteract the resolution of established fibrosis and promote disease progression. If so, it seems reasonable that clearance of CS cells could be beneficial for the treatment of IPF or at least for attenuation of its progression. Although further studies are warranted, our findings support the notions that cellular senescence could be an important player in IPF, and serve as a bridge, connecting lung aging and its quite frequent outcome – pulmonary fibrosis.

## MATERIALS AND METHODS

### Lung biopsies

As part of the investigative framework of the large collaborative EU project RESOLVE (2), enabling a systematic analysis of 17 different wound healing conditions, 30 patients with proven histopathology of UIP and a clinical diagnosis of IPF underwent combined clinical and biological analysis. This included sequential pulmonary function tests (PFT) and taking of lung biopsies during repeated bronchoscopies. Classification of patients as either limited or advanced UIP was based on the progress of disease in lung function using PFT values currently approved for assessment of IPF progression (forced vital capacity, FVC; diffusing capacity for carbon monoxide, DLCO) [42]. Biopsies for cell cultures were taken from five male patients with advanced UIP/IPF lacking signs of active bronchitis. Mean age was 63.9 ± 11.6 years. Transbronchial lung biopsies were removed under radiological control from two separate locations of either the left or right lower lobes in accordance with current findings in high resolution-computed tomography scans. All procedures were approved by the Ethical Committee of the Medical University of Vienna (http://ClinicalTrials.gov Identifier: NCT01687946).

### Cell cultures

Two transbronchial lung specimens were cut into approximately 2×2×2 mm cubes immediately after removal, and were transferred into a cell culture flask and allowed 5-10 min for adhesion. Cell cultures were incubated at 37°C, 5% CO_2_ in mesenchymal growth medium until reaching confluence. Primary cultures of human pulmonary fibroblasts from both IPF patients and normal cells (obtained from ScienCell, Carlsbad, CA, US; CAT# 3300) were grown in standard conditions in Dulbecco's modified Eagles medium (DMEM) (Biological Idustries, Beit Haemek, Israel). Cells were passaged upon 75-80% confluence and counted. Senescence was induced either by serial passaging (for replicative CS) or by H_2_O_2_ (Sigma Aldrich, St. Louis, MO, USA) treatment for 2 hours at varying concentrations (for stress-induced CS) in FCS-free DMEM. Cell survival and cytotoxicity was measured by neutral red assay.

### SA-β-galactosidase assay and immunostaining

The SA-β-gal assay was carried out using the Sigma Aldrich SA-β-galactosidase detection kit, according to the manufacturer's protocol. α-SMA was detected as previously described [8] using one μl of Monoclonal Anti-Actin, α-Smooth Muscle Alkaline Phosphatase (AP)-conjugated antibody (α-SMA). Immunostained plates were visualized using the Olympus IX2 series or PrimoVert microscopes.

### Confocal microscopy and ImageStreamX analysis

Twenty thousand cells were seeded in Ibidi 8-well slides in 200 μl of complete medium and incubated for 24 hours at 37 °C and 5% CO_2_. DiD (2.85 μM) in serum-free DMEM (Biological Industries) was added per well. Cells were incubated for 30 min followed by two washes with PBS containing Ca & Mg, fixated with 4% PFA for 20 min in room temperature, and then washed twice with PBS. Blocking of non-specific binding with 5% BSA for 30 min was performed, and again washed with PBS. Five μl of Phalloidin stock solution was diluted in 200 μl PBS with 1% BSA for each well. One μl of DAPI was added per each well (final concentration of 10 μg/ml). After 20 min of incubation, wells were mounted with 200 μl PFN. To avoid photobleaching, all the above procedural steps were performed in the dark. Cells were visualized using the Olympus IX81 confocal microscope. For ImageStreamX analysis, the cells were suspended in 1.5-ml Eppendorfs, and the same protocol was used for staining with fluorescent dyes.

### Statistical evaluation

The statistical package for the social sciences (SPSS, Inc., Chicago, IL) software was used for the statistical evaluation of the results. Statistical evaluation was carried out using factorial analysis (ANOVA) to test for differences between the control and the experimental groups. Values of p < 0.05 were considered statistically significant. Statistical analysis of ImageStreamX results was performed with the IDEAS® software (Amnis, Millipore, Billerica, MA, USA) according to their guidelines.
