# Erratum: Cellular senescence-like features of lung fibroblasts derived from idiopathic pulmonary fibrosis patients

**Published:** 2015-09-22

**Authors:** Hagai Yanai, Albert Shteinberg, Ziv Porat, Arie Budovsky, Alex Braiman, Rolf Zeische, Vadim E. Fraifeld

**Aging (Albany NY) 2015; 7(9)**: **664-672**.

PMCID: PMC 4600624 PMID: 26399448

The last name of sixth author Rolf Zeische is misspelled. The correct name is **Rolf Ziesche**.

